# Distinct Effects of Nalmefene on Dopamine Uptake Rates and Kappa Opioid Receptor Activity in the Nucleus Accumbens Following Chronic Intermittent Ethanol Exposure

**DOI:** 10.3390/ijms17081216

**Published:** 2016-07-27

**Authors:** Jamie H. Rose, Anushree N. Karkhanis, Björn Steiniger-Brach, Sara R. Jones

**Affiliations:** 1 Department of Physiology and Pharmacology Wake Forest School of Medicine, Winston-Salem, NC 27157, USA; jrose9@elon.edu (J.H.R.); akarkhan@wakehealth.edu (A.N.K.); 2 H. Lundbeck A/S, Ottiliavej 9, 2500 Valby, Denmark; bsbr@lundbeck.com

**Keywords:** C57BL/6, mouse, voltammetry, release, partial agonist, dynorphin, dopamine, alcohol

## Abstract

The development of pharmacotherapeutics that reduce relapse to alcohol drinking in patients with alcohol dependence is of considerable research interest. Preclinical data support a role for nucleus accumbens (NAc) κ opioid receptors (KOR) in chronic intermittent ethanol (CIE) exposure-induced increases in ethanol intake. Nalmefene, a high-affinity KOR partial agonist, reduces drinking in at-risk patients and relapse drinking in rodents, potentially due to its effects on NAc KORs. However, the effects of nalmefene on accumbal dopamine transmission and KOR function are poorly understood. We investigated the effects of nalmefene on dopamine transmission and KORs using fast scan cyclic voltammetry in NAc brain slices from male C57BL/6J mice following five weeks of CIE or air exposure. Nalmefene concentration-dependently reduced dopamine release similarly in air and CIE groups, suggesting that dynorphin tone may not be present in brain slices. Further, nalmefene attenuated dopamine uptake rates to a greater extent in brain slices from CIE-exposed mice, suggesting that dopamine transporter-KOR interactions may be fundamentally altered following CIE. Additionally, nalmefene reversed the dopamine-decreasing effects of a maximal concentration of a KOR agonist selectively in brain slices of CIE-exposed mice. It is possible that nalmefene may attenuate withdrawal-induced increases in ethanol consumption by modulation of dopamine transmission through KORs.

## 1. Introduction

Chronic alcohol use disorders are an enormous economic and financial burden in the United States [1]. A large body of literature has shown that chronic alcohol exposure down-regulates dopamine transmission in the nucleus accumbens (NAc) mice: [2]; rats: [3,4]; humans: [5,6], potentially leading to the negative affective states experienced during withdrawal [7,8]. It is plausible that attenuated dopamine neurotransmission following chronic ethanol exposure and withdrawal may be driven, at least in part, by increased function of inhibitory receptors on dopamine terminals in the NAc [2,9,10,11].

The dynorphin/Kappa opioid receptor (KOR) system is of particular interest due to its involvement in modulating ethanol drinking behaviors. For example, prodynorphin knockout mice showed lower preference for ethanol and consumed lower amounts of ethanol compared to wild-type mice [12]. Furthermore, multiple studies have shown increases in NAc KOR function following chronic ethanol exposure [9,10,13,14]. In fact, augmented NAc KOR function may mediate CIE-induced reductions in dopamine terminal function and increased ethanol consumption following extended ethanol exposure and withdrawal [15,16]. Furthermore, intra-NAc KOR blockade reduces relapse-like drinking behavior in rodents [16]. These data suggest that the dynorphin/KOR system may be a promising pharmacotherapeutic target to reduce relapse drinking in patients with alcohol dependence.

Nalmefene (6-methylene naltrexone) is a pharmacotherapeutic agent approved in the European Union to combat heavy alcohol drinking in at-risk patients [17] (25 February 2013). Preclinical work showed that intra-NAc infusions of nalmefene reduced ethanol intake at lower doses in ethanol-dependent ethanol self-administering rats than in non-dependent animals [16], an effect that was credited to the partial agonist activity and high affinity of nalmefene at KORs [16,18]. Despite this behavioral evidence, the effects of nalmefene on dopamine terminal and NAc KOR function following chronic ethanol exposure are poorly understood, and elucidation of its effects may aid in understanding the basis of its clinical efficacy. To this end, ex vivo fast scan cyclic voltammetry (FSCV) was used to evaluate the effects of nalmefene on dopamine transmission and KOR function in the NAc core, 72 h following five weeks of chronic intermittent ethanol (CIE) exposure, in male C57BL/6 (C57) mice.

## 2. Results

### 2.1. CIE Exposure Reduced Dopamine Transmission in the NAc Core

**Figure 1:**
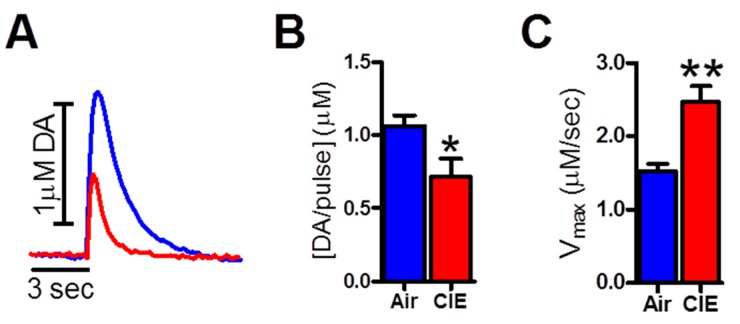
Chronic intermittent ethanol (CIE) exposure reduced dopamine release and increased dopamine uptake in the nucleus accumbens (NAc) core. Representative FSCV traces are overlaid in (**A**) (Air: blue trace; CIE: red trace); (**B**) CIE reduced dopamine release in brain slices of the NAc from CIE-exposed mice compared to air-exposed controls; (**C**) CIE increased dopamine uptake rates (*V*_max_) in brain slices from CIE-exposed mice compared to controls. * *p* < 0.05, ** *p* < 0.05 (CIE: chronic intermittent ethanol).

We maintained BECs at behaviorally and physiologically relevant levels (219.50 ± 39.31 mg/dL), as per [19]. Representative FSCV traces are overlaid in Figure 1A (Air: blue trace; CIE: red trace). Consistent with previous work from our laboratory [2,9] CIE reduced dopamine release (Figure 1B, *t*_18_ = 2.38 *p* < 0.05; Air: 1.06 ± 0.26 µM; CIE: 0.71 ± 0.40 µM) and increased rates of dopamine uptake (Figure 1C, *t*_17_ = 3.80, *p* < 0.05; Air: 1.52 ± 0.32 µM/s; CIE: 2.46 ± 0.68 µM/s) compared to air-exposed controls.

### 2.2. Nalmefene Slowed Dopamine Uptake Rates More in Brain Slices from CIE-Exposed Mice Than Controls

**Figure 2:**
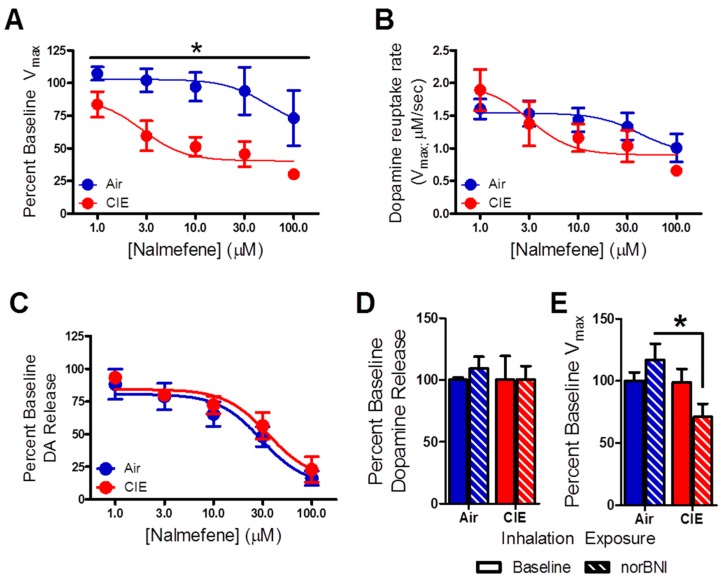
Nalmefene attenuates uptake rates in brain slices from CIE-exposed mice. (**A**) Nalmefene concentration-dependently attenuated the maximal rate of uptake rate (*V*_max_) in both groups, although this effect was greater in brain slices from CIE exposed animals compared to air-exposed mice; (**B**) nalmefene reduced the uptake rate in CIE-exposed animals to a level comparable to that found in air-exposed mice even; (**C**) nalmefene concentration-dependently decreased dopamine release similarly in the inhalation groups; (**D**) the KOR antagonist, norbinaltorphimine (norBNI) did not alter dopamine release in either inhalation group; (**E**) NorBNI reduced uptake rates in brain slices from CIE-exposed mice compared to controls. * *p* < 0.05 (CIE: chronic intermittent ethanol).

To examine the effects of nalmefene on dopamine release and uptake following ethanol vapor and air exposure, increasing concentrations of this compound were bath-applied to accumbal brain slices. A comparison of percent change in dopamine uptake rate showed that nalmefene dose-dependently reduced uptake rates in both groups (Figure 2A, *F*_4,8_ = 5.29, *p* < 0.01), although this effect was greater in CIE exposed animals (*F*_1,8_ = 7.94, *p* < 0.05). No interaction between these factors was detected (*F*_4,8_ = 5.40, *p* > 0.05). When the absolute values of dopamine uptake rates after application of nalmefene were compared across the two groups, there were no differences (Figure 2B). Moreover, two-way RM ANOVA revealed a main effect of nalmefene concentration on dopamine release (Figure 2C, *F*_4,9_ = 48.34, *p* < 0.001), which was similar between inhalation groups (*F*_1,9_ = 7.27, *p* > 0.05).

A separate group of brain slices were incubated in norBNI for 60 min to assess the effects of KOR blockade on dopamine transmission. A two-way ANOVA showed no effect of norBNI on dopamine release (Figure 2D, *F*_1,24_ = 0.12, *p* > 0.05), while a main effect of inhalation treatment on uptake rates (Figure 2E, *F*_1,26_ = 4.40, *p* < 0.05) was detected. Bonferroni post hoc analysis revealed a significant difference between the effects of norBNI on CIE-exposed mice compared to air-exposed controls (*p* < 0.05).

### 2.3. Nalmefene Reversed the Dopamine-Decreasing Effects of U50,488 in CIE-Exposed Mice

**Figure 3:**
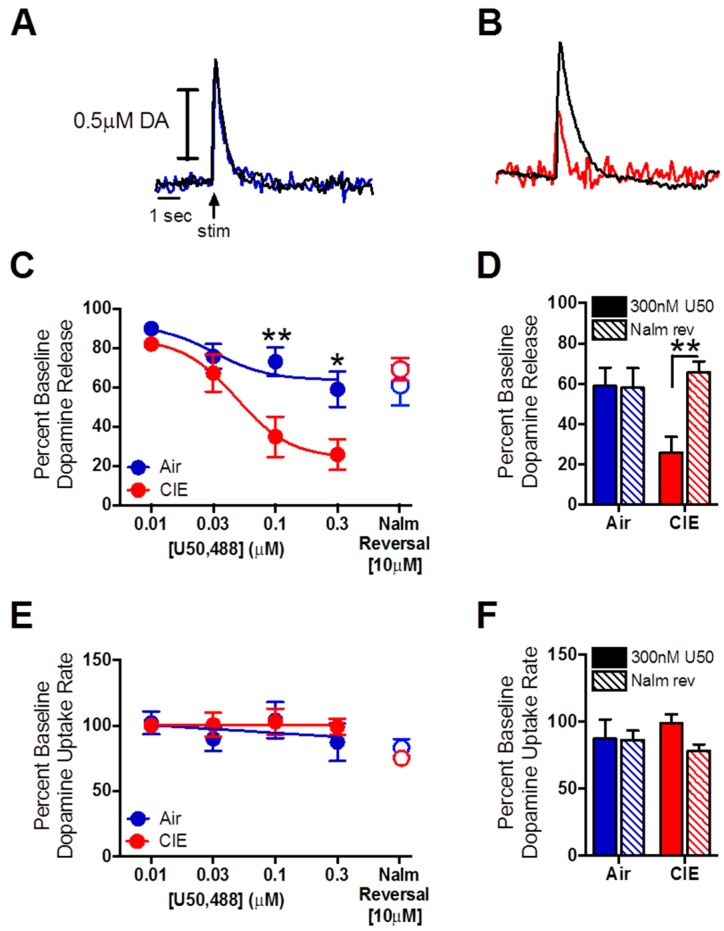
Nalmefene reversed the dopamine-decreasing effects of U50,488 in CIE-exposed mice. (**A**) Representative traces of the effects of 0.3 µM U50,488 on dopamine release in brain slices from air (solid blue) and (**B**) CIE (solid red) exposed mice. The black line (overlaid) represents the effect of 10.0 µM nalmefene on these signals; (**C**) KOR activation with U50,488 dose-dependently reduced dopamine release more in brain slices from CIE-exposed mice compared to controls; (**C**,**D**) 10 µM nalmefene restored dopamine release following a maximal concentration of the KOR agonist U50,488 in brain slices from CIE-exposed mice to control levels; (**E**,**F**) U50,488 did not alter dopamine uptake rates across the concentration response curve, and nalmefene had no additional effect on uptake rates in either inhalation group. * *p* < 0.05, ** *p* < 0.01. (CIE: chronic intermittent ethanol; KOR: κ opioid receptor).

Representative traces showing the effects of 0.3 µM U50,488 on dopamine release and uptake (Figure 3A, blue, air; Figure 3B, red, CIE) and 10.0 µM nalmefene reversal (black line, overlaid). The effects of CIE on KOR function were examined with increasing concentrations of U50,488. A RM two-way ANOVA revealed a main effect of KOR activation on dopamine release that was dose-dependent (Figure 3C, *F*_3,10_ = 31.69, *p* < 0.001), which was greater in brain slices from mice exposed to CIE (*F*_1,10_ = 6.26, *p* < 0.05). An interaction between these factors was also detected (*F*_3,10_ = 5.51, *p* < 0.01). Bonferroni post hoc analysis revealed a significant difference between inhalation groups at the 0.1 µM (*p* < 0.01) and 0.3 µM (*p* < 0.05) U50,488 concentrations.

As nalmefene is a partial KOR agonist, it competes for the endogenous ligand binding site on the receptor [18,20]. To determine the ability of nalmefene to reverse the dopamine-decreasing effects of KOR activation with an exogenous ligand, 10.0 µM of the compound was added to the bath solution following the 0.3 µM concentration of U50,488. A two-way ANOVA, with U50,488 and nalmefene as factors revealed a main effect of drug (U50,488 vs. nalmefene; Figure 3D, *F*_1,10_ = 5.67, *p* < 0.05), as well as an interaction between drug and inhalation treatment (*F*_1,10_ = 6.19, *p* < 0.05). An effect of inhalation treatment alone was not detected (*F*_1,10_ = 2.46, *p* > 0.05). Bonferroni post hoc analysis revealed a significant increase in dopamine release due to nalmefene reversal in the CIE-exposed group compared to the 0.3 µM U50,488 concentration (*p* < 0.01).

As nalmefene reduced dopamine uptake rates to a greater extent in brain slices from CIE-exposed mice compared to air-exposed controls, we examined the effects of a nalmefene challenge on U50,488-induced reductions in dopamine uptake rates. Surprisingly, a two way RM ANOVA revealed no effect of U50,488 on dopamine uptake rates (Figure 3E, *F*_3,8_ = 1.26, *p* > 0.05) in either inhalation group (*F*_1,8_ = 0.17, *p* > 0.05). Further, a two-way RM ANOVA revealed that nalmefene had no effect on dopamine uptake rates following the maximal (0.3 µM) concentration of U50,488 (Figure 3F, *F*_1,16_ = 0.04, *p* > 0.05).

## 3. Discussion

**Figure 4:**
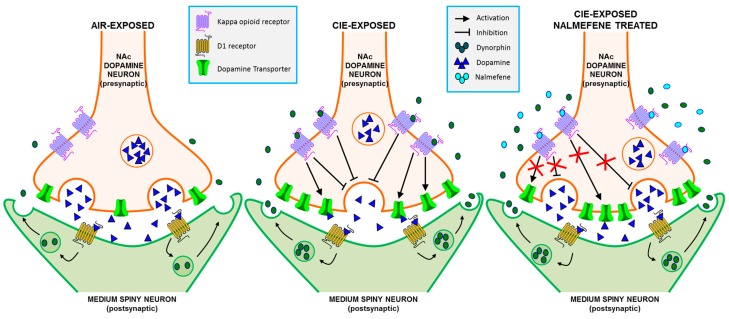
Schematic diagram of chronic intermittent ethanol (CIE)-induced changes in kappa opioid receptors (KOR), dopamine transporters (DAT), dopamine release, and dynorphin and nalmefene effects on KOR function based on the current hypotheses. In comparison to air-exposed mice (**Left**), CIE-exposed mice (**Middle**) have lower dopamine release and greater uptake rates. This is potentially caused by increased KOR responses, as KORs inhibit dopamine release and increase DAT function when activated. The increased responses could be caused by increased levels of endogenous dynorphin released from the postsynaptic medium spiny neurons, an increase in receptor expression on the membranes of the presynaptic dopamine neurons, or both. (**Right**) shows a synapse from CIE-exposed animals in the presence of nalmefene. Nalmefene slows the KOR agonist-induced augmentation of uptake rates and inhibits the reduction in dopamine release in CIE-exposed animals, thus normalizing the dopamine release and uptake.

The present study aimed to discern the pharmacological effects of nalmefene on dopamine terminal and KOR function following CIE exposure in mice (Figure 4). Congruent with previous work [2,3,4,9,11], CIE exposure reduced dopamine release, augmented rates of dopamine uptake and increased κ opioid system sensitivity to an agonist, promoting a hypodopaminergic state of the NAc. Nalmefene concentration-dependently reduced dopamine release similarly between inhalation conditions, but attenuated uptake rates more in brain slices from CIE-exposed mice compared to controls. Additionally, we found that a single concentration of nalmefene reversed the dopamine-decreasing effects of a maximal concentration of the KOR agonist U50,488 selectively in brain slices from CIE-exposed mice. These data are the first to demonstrate dopamine terminal modulation by nalmefene and point to mechanisms that may underlie nalmefene-induced reductions in ethanol intake following CIE exposure [15,16].

### 3.1. Nalmefene Reduced Dopamine Release Equally in Both Inhalation Groups, but Attenuated Dopamine Uptake Rates More in Brain Slices of CIE-Exposed Mice

Our data show similar dopamine-decreasing effects of nalmefene between inhalation groups, indicating that dynorphin tone may not be present in striatal brain slices. If dynorphin tone were to be present, nalmefene would compete with the endogenous ligand for receptor occupancy [21], resulting in antagonist-like effects, which could be measured voltammetrically as an increase in stimulated dopamine release [22]. Notably, control experiments using norBNI in this study showed no effect of KOR blockade on dopamine release in either inhalation group, further suggesting that dynorphin tone is not measurable in brain slices with the current technique. Therefore, it seems that nalmefene was acting as an agonist at KORs to reduce dopamine release [18].

In addition to being a partial KOR agonist, nalmefene also has antagonistic activity at mu (MOR) and delta (DOR) opioid receptors [18], making definitive designation of its effects to any one opioid receptor difficult. It has been shown using in vivo microdialysis that local activation of DORs and MORs results in an increase in extracellular dopamine [23]. However, DORs do not directly modulate dopamine afferents [24]. In fact, DOR activation-induced efflux of dopamine has been shown to occur via a mechanism involving the glutamate system as blockade of *N*-methyl-d-aspartate receptors in the presence of DOR agonist inhibited dopamine release [25]. Therefore, a direct DOR-driven modulation of presynaptic dopamine transmission is unlikely. Inhibitory MORs are primarily localized to GABAergic interneurons that feed onto dopamine terminals [26] and, therefore, it is possible that the dopamine decreasing effect of nalmefene observed in the current study could be due to a combination of MORs and KOR activity. However, a previous study showed that a MOR agonist reduced dopamine release evoked by single pulse stimulation, while increasing dopamine release evoked by phasic stimulation [27]. Since we used single pulse electrical stimulations in the current study and nalmefene is a MOR antagonist, it is unlikely that the reduction in dopamine release is via a mechanism involving the MORs. Overall however, nalmefene modulation of DORs/MORs, and its influence on dopamine transmission is not known in this context and some contribution of these receptors to the present findings cannot be completely ruled out.

Although the effects of nalmefene on dopamine release were similar between inhalation groups, this compound concentration-dependently reduced dopamine uptake rates more in brain slices from CIE-exposed mice compared to controls. In this instance, it appears as though nalmefene is acting to reduce dopamine release and uptake through KORs. Notably, dopamine transporters have been consistently reported to be functionally upregulated following CIE exposure [2,3,4,9]. Here, we found that KOR blockade slowed uptake rates in brain slices from CIE-exposed mice compared to air-exposed controls using a single concentration of norBNI, which may be due to its documented effects on KOR-related intracellular signaling cascades [28]. Dopamine uptake has been previously reported to be altered by KOR agonists, given either acutely [29] or chronically [30,31]. A recent study showed that KORs exist both independently and in complex with dopamine transporters, and regulate dopamine transporter function via an ERK1/2-dependent pathway [29]. Therefore, we hypothesize that the physical or functional connection between dopamine transporters and KORs [29] is fundamentally altered following CIE, driving KOR antagonist-mediated reductions in uptake rates in the present experiments. However, using voltammetric methods in slices, we also found that KOR activation did not augment [29,30] or attenuate [32,33] dopamine uptake rates as reported previously, but eliminated nalmefene-induced reductions in dopamine uptake. Additional investigations into the interplay of U50,488 and nalmefene on KOR-induced alterations in dopamine uptake rates are needed to fully elucidate these findings.

### 3.2. Dopamine Release Is Restored by Nalmefene Following KOR Activation in Brain Slices from CIE-Exposed Mice

To better understand the distinct effects of nalmefene on KOR function between the inhalation groups, a high concentration of nalmefene was applied to brain slices following a maximal concentration of U50,488. As earlier experiments suggested that dynorphin tone is undetectable in brain slices, the addition of U50,488 was necessary to examine any antagonistic effects of nalmefene on KORs. We found that nalmefene reversed the dopamine-decreasing effects of U50,488 in brain slices from CIE-exposed mice, suggesting that nalmefene competed with U50,488 for KOR occupancy. It is possible that this effect is due to CIE-induced receptor upregulation or functional supersensitivity of the kappa opioid system compared to air-exposed animals. In other work, CIE exposure increased levels of KOR binding in seizure-resistant mice [34] and augmented dynorphin-stimulated KOR activity in Wistar rats [13] compared to controls. Therefore, it is plausible that the observed reversal of the dopamine-decreasing effects of U50,488 in brain slices from CIE-exposed mice could be due to the antagonist capabilities of nalmefene interacting with the physical or functional upregulation of KORs.

### 3.3. Comparison of norBNI and Nalmefene

Compounds that alleviate ethanol-induced reductions in dopamine transmission following chronic ethanol exposure, such as those that manipulate KORs, may assuage withdrawal symptoms. Previous work has shown that norBNI blocks withdrawal-induced increases in brain reward thresholds following chronic ethanol [7] and cocaine [35] exposure, and reduces ethanol withdrawal-induced increases in drinking via systemic [9,15] and intra-NAc [16] administration in rodents. Although one study showed increased ethanol intake in animals following norBNI administration [36] it should be noted that rats in that study had continuous access to ethanol and did not undergo withdrawal, unlike studies that show a norBNI-induced reduction in ethanol intake in intermittently exposed, dependent animals [9,15,16]. Moreover, conditioned place preference was absent in mice lacking the prodynorphin gene and mice administered norBNI [37]; however, in that study, conditioned place preference was not tested in a model of alcohol dependence and the mice likely did not experience withdrawal. These data indicate that KOR blockade alleviates withdrawal-induced reductions in dopamine system function in a direct and behaviorally-relevant manner. It is possible that norBNI induces these changes by reducing the effects of endogenous dynorphin on KORs or attenuating the intrinsic activity of functionally upregulated KORs [9,10,11], present work. For example, norBNI has been shown to reverse the low-dopamine state of the animals exposed to chronic stress, which then potentially results in a reduction in ethanol intake [38]. Similar to norBNI, systemic [15] and intra-NAc [16] nalmefene reduces withdrawal-induced increases in ethanol drinking in rodents and drinking in alcoholics [39,40]. As a partial agonist, nalmefene provides antagonist-like effects in the presence of a full agonist [21]. The ability of nalmefene to reverse the dopamine decreasing effects of U50,488 in brain slices from CIE-exposed mice compared to controls is likely innate to an upregulation in KOR function following CIE exposure [9,10,11], present work. Secondly, nalmefene reduced dopamine uptake to a greater extent in brain slices from CIE exposed animals. We hypothesize that CIE alters downstream signaling or physical interactions between receptors and dopamine transporters, as reported previously [29]. In summary, it is likely that norBNI is effective by increasing tonic levels of dopamine at baseline while nalmefene is effective via a reduction in dopamine uptake.

### 3.4. Behavioral Implications of the Effects of Nalmefene on Dopamine Terminal Function

Intra-cerebroventricular [15] and intra-accumbal [16] nalmefene reduces ethanol intake at lower doses in ethanol dependent animals, compared to non-dependent animals, in part due to its high affinity and the unique action of this compound on KORs. It is plausible that nalmefene-induced reductions in ethanol drinking may be due to reductions in ethanol withdrawal-induced hypodopaminergia via attenuated uptake rates and increased dopamine release, selectively in brain slices from CIE-exposed mice ex vivo. In fact, dopamine transporter knockout mice, with inherently reduced rates of dopamine clearance compared to wild-type mice [41] consume less ethanol than their wild-type and heterozygous counterparts [42], providing evidence to support this hypothesis.

The time-course of nalmefene-induced alterations in neurobiology is rapid and beneficial in the therapeutic application of this compound. In fact, ethanol intake in ethanol dependent rats was reduced with lower doses of nalmefene than non-dependent animals [15], and occurred with administration approximately 30 min prior to ethanol self-administration testing. In fact, one study showed that nalmefene was absorbed within one hour of administration in healthy subjects following single and multiple dosing schedules [43]. Thus, it is not surprising that alcohol dependent individuals with high or very high drinking risk level who are prescribed nalmefene take the compound orally when they predict they will encounter a high-risk situation (i.e., social environments where alcohol may be present), and consistently report reductions in overall alcohol consumption [40,44,45,46]. Use of nalmefene on a continuous schedule dose-dependently reduces alcohol intake over time [39,47] and attenuates relapse to heavy drinking [40,47] in treatment-seeking patients with alcohol dependence. Together, preclinical and clinical evidence strongly suggest that nalmefene reduces ethanol/alcohol intake on a rapid timescale, and data presented here suggest that KOR modulation of dopamine transmission contributes to the behavioral effects of systemic nalmefene administration.

## 4. Experimental Procedures

### 4.1. Subjects

Male C57 mice (6–8 weeks old, Jackson Labs, Bar Harbor, ME, USA) were used for all experiments. Animals were allowed at least one week of habituation to the housing environment before CIE procedures began. All mice were individually housed and maintained on a 12-h light-dark cycle (lights off at 14:00), with a red-room light illuminated during the animals’ dark cycle. Standard rodent chow and water were available ad libitum, and replaced daily in the CIE exposed group. At 6–8 weeks old, the mice used in the present study are within the range of early adulthood [48,49]. Notably, mice ages considerably across the five weeks of air/CIE exposure (mice were 11–13 weeks of age at the time of sacrifice for ex vivo voltammetry experiments; both procedures are explained in detail below). At the time of sacrifice, mice are sexually mature and can be considered mature adults [48]. It is plausible that CIE exposure would result in differential neurobiological changes in young versus adult or old mice, but since the mice in this study are all considered “adult” in the literature, the differences are likely minimal. Animals were cared for according to the National Institutes of Health guidelines in Association for Assessment and Accreditation of Laboratory Animal Care, and all experimental protocols were approved by the Institutional Animal Care and Use Committee at Wake Forest University School of Medicine.

### 4.2. Chronic Intermittent Ethanol (CIE) Exposure

This study utilized a CIE exposure protocol previously published [9]. Briefly, mice were exposed to ethanol vapor (CIE) or room air for 16 h/day, followed by 8 hours of room air. This procedure was repeated four times before a 72-h abstinence period (one cycle). Cycles were repeated five times. Although alterations in dopamine transmission are observed after a minimum of three cycles of exposure, the current study used five cycles of exposure to maintain continuity with a set of experiments examining the behavioral effects of KOR system manipulation following CIE exposure [9]. Approximately 30 min prior to chamber start time (17:00), air inhalation mice were systemically injected with pyrazole (1.0 mmol/kg; i.p.; Sigma-Aldrich, St. Louis, MO, USA), an alcohol dehydrogenase inhibitor, mixed with saline. Similarly, CIE exposure mice were systemically injected with pyrazole (1.0 mmol/kg; i.p.) mixed with ethanol (1.6 g/kg; i.p.). Mice metabolize ethanol very rapidly, and a combination of pyrazole, a loading dose of ethanol and continuous ethanol vapor exposure is required to maintain blood ethanol concentrations BECs in the desired range for 16 h in the ethanol chamber. BECs were measured the mornings following the first and final inhalation exposure of each cycle. Even though CIE exposure is a non-contingent method, it has been shown to drive augmented compulsive/anxiety-like behavior and an escalation of ethanol drinking, which suggests that ethanol dependence is achieved [9,50]. On the other hand, the other methods of alcohol exposure, such as drinking in the dark, do not usually produce dependence, which is routinely observed in human alcoholics.

### 4.3. Blood Ethanol Concentration (BEC) Measurement

To ensure proper ethanol chamber function and physiologically relevant BECs, a submandibular vein blood draw was performed in ethanol vapor-exposed mice only. Less than 15 µL of blood was collected in BD microtainer tubes lined with lithium heparin (Becton Dickinson and Company, Franklin Lakes, NJ, USA). For BEC measurement, standards and samples were prepared with a commercially available alcohol dehydrogenase assay (Carolina Liquid Chemistries Corporation, Brea, CA, USA) and carefully pipetted into a 96 well plate. Plate analysis was done with SoftMax Pro Software, version 5 (Molecular Devices Corporation, Sunnyvale, CA, USA). Mean BECs were 219.50 ± 39.31 mg/dL.

### 4.4. Ex Vivo Fast Scan Cyclic Voltammetry (FSCV)

FSCV was used to detect CIE-induced changes in dopamine release and uptake, as well as the effects of nalmefene, U50,488, and nor-binaltorphimine (norBNI), a KOR-specific agonist and antagonist, respectively, on dopamine dynamics and kinetics. Briefly, 300-µm-thick coronal brain slices, containing the NAc core were, prepared using a vibrating tissue slicer. Slices were incubated in oxygenated artificial cerebrospinal fluid and heated to 32 °C for approximately 60 min prior to experiment start. A bipolar stimulating electrode (Plastics One, Roanoke, VA, USA) and carbon fiber microelectrode (≈50 µm length, 7 µm radius (Goodfellow Corporation, Berwyn, PA, USA) were placed within 100 µm of each other on the surface of the slice. Dopamine efflux was induced by a single, rectangular, electrical pulse (4.0 ms; 350 µA, monophasic; interstimulus interval: 180 s), and detected by applying a triangular waveform every 100 ms to the recording electrode (−0.4 to +1.2 to −0.4 V vs. Ag/AgCl, 400 V/s). When baseline collections were stable for three consecutive stimulations, nalmefene (1.0–100.0 µM, generously provided by H. Lundbeck A/S), norBNI (1.0 µM, graciously provided by National Institute on Drug Abuse, NIDA) and U50,488 (0.01–0.3 µM, generously provided by NIDA) were cumulatively added to the bath. Following the maximal concentration of U50,488 (0.3 µM), a challenge concentration of nalmefene (10.0 μM) was added to the bath solution. In a separate set of experiments, a single dose of norBNI (1.0 µM) was applied to brain slices to identify the effects of KOR blockade on dopamine release and uptake measures. Clear current versus time plots were obtained using background current subtraction methods. Electrodes were calibrated immediately after experiments by recording their response (in nA) to a known concentration of dopamine (3.0 μM) using a flow-injection system.

### 4.5. Data Analysis

Representative pre-drug traces of electrically-stimulated dopamine release were individually modeled. Data were collapsed across inhalation groups to obtain baseline dopamine release and uptake measures. Dopamine release was calculated as the amount of electrically-evoked dopamine released per stimulation, and *V*_max_ was calculated as the maximal rate of uptake at the dopamine transporter. The apparent affinity of dopamine for the dopamine transporter (apparent *K*_m_) remained constant at 160 nM throughout analysis [51]. The effects of KOR ligands on dopamine release and uptake parameters were similarly analyzed. Demon Voltammetry and Analysis software [52] was used to collect and analyze all data.

Graphs were created and statistical analyses were applied with GraphPad Prism (version 5, La Jolla, CA, USA). Student’s *t*-tests were used to analyze the effects of inhalation exposure on dopamine release and uptake rates. Repeated measures (RM) two-way analysis of variance (ANOVA) was used to determine the effects of increasing concentrations of U50,488 and nalmefene on dopamine release and uptake, with inhalation exposure and drug concentration as factors. Additionally, non-RM two-way ANOVAs, with inhalation exposure and drug concentration as factors, were used to determine the ability of nalmefene to reverse the dopamine-decreasing effects of the 0.3 µM U50,488 concentration, the effects of nalmefene on the maximal rate of uptake following 0.3 µM U50,488, as well as the effects of 1.0 µM norBNI on dopamine release and uptake parameters. When a significant main effect was detected, Bonferroni post hoc analysis was applied.

## 5. Conclusions

Due to the high rate of recidivism to alcohol use disorders, the need for effective pharmacotherapies for this disorder is high. Modulation of KORs to restore dopamine system function during alcohol withdrawal is of interest therapeutically [53]. Overwhelming preclinical behavioral evidence [15,16] and clinical work in patients with alcohol dependence [39,40,44,45,47] indicate favorable therapeutic effects of nalmefene on alcohol consumption. Notably, the effects of nalmefene on drinking are due, in part, to its high affinity and partial agonist activity of this compound on KORs, particularly in the NAc [16]. We report that nalmefene reduced dopamine uptake rates and reversed the dopamine-decreasing effects of KOR activation, suggesting that nalmefene may augment dopamine transmission in vivo (Figure 4). Increased accumbal dopamine transmission by nalmefene would attenuate the hypodopaminergic state of this region through reductions in KOR activity and dopamine uptake rates. These mechanisms may underlie nalmefene-induced reductions in ethanol intake via increased dopaminergic function rodents: [9,15,16]; humans: [39,40,44,45,47]. These data promote insight into the pharmacological effects of nalmefene, and may provide a better understanding of its clinical efficacy.
